# The roles and involvement of global health partners in the health workforce: an exploratory analysis

**DOI:** 10.1186/s12960-023-00825-5

**Published:** 2023-05-24

**Authors:** Andrea Nove, Onyema Ajuebor, Khassoum Diallo, James Campbell, Giorgio Cometto

**Affiliations:** 1 Novametrics Ltd, Derby, DE56 4HQ United Kingdom; 2 World Health Organization, Health Workforce Department, Geneva, Switzerland

**Keywords:** Health workforce, Human resources for health, Health workforce assessment, Information exchange, Health labour market

## Abstract

Development partners and global health initiatives are important actors in financing health systems in many countries. Despite the importance of the health workforce to the attainment of global health targets, the contribution of global health initiatives to health workforce strengthening is unclear. A 2020 milestone in the *Global Strategy on Human Resources for Health* is that “all bilateral and multilateral agencies have participated in efforts to strengthen health workforce assessments and information exchange in countries.” This milestone exists to encourage strategic investments in the health workforce that are evidence-based and incorporate a health labour market approach as an indication of policy comprehensiveness. To assess progress against this milestone, we reviewed the activities of 23 organizations (11 multilaterals and 12 bilaterals) which provide financial and technical assistance to countries for human resources for health, by mapping grey and peer-reviewed literature published between 2016 and 2021. The *Global Strategy* states that health workforce assessment involves a “deliberate strategy and accountability mechanisms on how specific programming contributes to health workforce capacity-building efforts” and avoids health labour market distortions. Health workforce investments are widely recognized as essential for the achievement of global health goals, and some partners identify health workforce as a key strategic focus in their policy and strategy documents. However, most do not identify it as a key focus, and few have a published specific policy or strategy to guide health workforce investments. Several partners include optional health workforce indicators in their monitoring and evaluation processes and/or require an impact assessment for issues such as the environment and gender equality. Very few, however, have embedded efforts in their governance mechanisms to strengthen health workforce assessments. On the other hand, most have participated in health workforce information exchange activities, including strengthening information systems and health labour market analyses. Although there is evidence of participation in efforts to strengthen health workforce assessments and (especially) information exchange, the achievement of this milestone of the *Global Strategy* requires more structured policies for the monitoring and evaluation of health workforce investments to optimize the value of these investments and contribute towards global and national health goals.

**Supplementary Information:**

The online version contains supplementary material available at 10.1186/s12960-023-00825-5.

## Background

Over the last two decades, the emergence of global health initiatives has changed the way that technical and financial support is provided for health. Their impact on health systems (including the health workforce)—and the need for methods and processes to monitor this impact—has often been overlooked [1–3]. Where the impact has been assessed, some positive impacts have been noted such as increased domestic production and improved retention [2]. However, concerns have been raised about a lack of coordination with national plans for health worker education, training and deployment, and labour market distortions due to, for example, differential pay scales [2, 3], which can weaken already fragile health systems. The health labour market is complex, with the result that health interventions do not always have the expected or desired effect [4]. The COVID-19 pandemic has highlighted this complexity and the importance of effective health workforce planning and management, including impact assessment [5].

The 2006 *World Health Report* drew attention to the magnitude of global health workforce challenges, and since its publication there has been increasing recognition of the importance of investing in the health workforce as part of efforts to achieve universal health coverage (UHC) [6]. In 2016, the World Health Assembly (WHA) adopted the *Global Strategy on Human Resources for Health* (HRH) [7], which sets out the policy agenda to ensure that the health workforce is adequate, well distributed and fit for purpose and thus allow for the attainment of UHC and the sustainable development goals. It calls on global health initiatives to support HRH capacity-building efforts by moving beyond disease-specific in-service training and incentives, and focusing on ensuring that investments in HRH are sustainable and do not inadvertently weaken health systems.

The *Global Strategy on HRH* established periodic reporting requirements for World Health Organization (WHO) Member States, facilitated by WHO. Milestone 4.3 states that by 2020, “*All bilateral and multilateral agencies have participated in efforts to strengthen health workforce assessments and information exchange in countrie*s”. This milestone exists to encourage strategic investments in the health workforce that are comprehensive and sustainable, and incorporate a health labour market approach to health workforce strengthening efforts.

The WHA resolution adopting the *Global Strategy* included three key actions relating to assessing the health workforce implications of health policy and programming: (1) partners should coordinate and align their investments in education, employment, health, gender, and labour in support of domestic financing aimed at addressing national health workforce priorities; (2) global health initiatives should ensure that all grants include an assessment of the health workforce implications, leverage national coordination and leadership, and contribute to efficient investment in and effective implementation of national health workforce policies; (3) an assessment should be made of the health workforce implications of technical resolutions brought before the WHA and WHO regional committees [8]. The *Global Strategy* states that health workforce assessment involves having a “*deliberate strategy and accountability mechanisms on how specific programming contributes to health workforce capacity-building efforts*”, and avoiding health labour market distortions.

The United Nations Secretary-General’s High-Level Commission on Health Employment and Economic Growth (ComHEEG) provided fresh impetus for countries to implement the *Global Strategy*. Health workforce data strengthening is one of the four key objectives of the *Global Strategy*, and one of ComHEEG’s recommendations related to the strengthening of health workforce data, information and accountability by using harmonized metrics and methodologies [9]. The WHA made commitments to act on the recommendations of the ComHEEG report, including HRH information exchange [10, 11].

The objective of this study was to identify and map grey and peer-reviewed literature which gives information about bilateral or multilateral agencies’ participation in efforts to strengthen health workforce assessments (in particular impact assessments) and information exchange. In particular, there was a focus on availability of the following types of document:

- policy and strategy documents which provide clear strategic direction for the organization’s HRH investments,
- documents indicating the extent to which health workforce assessments and/or HRH indicators are part of an organization’s routine monitoring and evaluation activities, and
- documents describing activities relating to improvements in HRH information exchange such as HRH information architecture and interoperability to enable better HRH advocacy, planning, policy-making, governance and accountability.

## Methods

This study maps the policies, strategies and activities of the 23 partner organizations listed in Table 1, as they relate to health workforce assessments and information exchange. These partners were identified by the authors from the WHO Health Workforce Department as being those most active in terms of investments in health systems strengthening and HRH initiatives (in terms of known or estimated volume of investments in HRH through either bilateral or multilateral channels), playing an active role in HRH policy dialogue and coordination mechanisms at either international or national levels, and having established communication channels with WHO. Some of the multilateral partners provide only technical support, some provide only financial support, and some do both. Within the table, they are organized according to their main type of activity in relation to the health workforce. To validate and clarify where necessary the findings from the textual analysis, each organization was invited to complete a questionnaire or speak with the lead author. Six organizations (marked with an asterisk in Table 1) responded to this request, between September and November 2021.

**Table 1:** Partner organizations included in the study

Multilaterals (*n* = 11)	Bilaterals (*n* = 12)
**Main role: funding support**	Australia
African Development Bank (AfDB)	Bill and Melinda Gates Foundation (BMGF)*
Asian Development Bank (ADB)	Canada
European Investment Bank (EIB)*	China
Gavi	European Commission (EC)
Global Financing Facility (GFF)	France
Global Fund*	Germany
World Bank	Japan
**Main role: technical support**	Norway
Organization for Economic Cooperation and Development (OECD)*	Saudi Arabia
United Nations Population Fund (UNFPA)	United Kingdom (UK)*
United Nations Children’s Fund (UNICEF)	United States of America (USA)
World Health Organization (WHO)*	

*These organizations provided additional information via a questionnaire or verbal interview

Our review focussed mostly on grey literature, because peer-reviewed literature on this type of activity is rare. Notwithstanding, a rapid and selective review of the peer-reviewed literature was conducted in addition.

The grey literature search was performed using the Google search engine, on various dates in the period 24 May 2021 to 22 June 2021 inclusive. A total of 24 searches was performed. Search terms included the partner organization’s name plus terms such as: “human resources for health”, “policy”, “technical guidance”, “assessment” and “data exchange” (see Annex for full details). These searches yielded hundreds of thousands of results. For each search, the first 50 results were reviewed, excluding any sponsored or promoted web pages, at which point the results became mostly duplicates or irrelevant. The reviewer clicked on the URL and briefly assessed the relevance of the piece to the objectives of this study. The URLs of items thought to be definitely or potentially relevant were saved for later full review. During the full review, if the item referred to other relevant items or the research team became aware of the publication of relevant new items, these were added to the review (“snowballing”) during the period July to December 2021.

The peer-reviewed literature search was conducted using the PubMed search engine. Search terms included the partner organization’s name plus the MeSH major topics “health workforce”, “staff development”, “workload”, “personnel staffing and scheduling”, plus terms such as “policy”, “measurement” and “impact assessment”(see Additional file1: Annex for full details). The first search yielded 163 articles, each of which was subjected to a title and abstract review to ascertain whether or not it met the inclusion criteria described below. The second search yielded 7 articles of which one was a duplicate, so 6 went forward for title and abstract review.

One researcher performed the initial review of the Google search results and the title and abstract review of peer-reviewed literature, to assess whether or not each item met the inclusion criteria. The inclusion criteria were (a) that the item was published after November 2016 (or it was published before this date but is the most recent document of its type and reflects current practice), (b) the item was written in English, (c) the item related to the activities of one or more of the organizations listed in Table 1 and (d) the item referred to:

- The partner organization’s policy or strategy on investments or programming which relate to HRH*, or
- The partner organization’s approach to assessment of HRH* impacts, or
- Ad hoc impact assessments on HRH* or other topics, even if no organizational policy or strategy exists, or
- The partner organization’s approach to HRH* information exchange between data holders and/or data sources, or
- HRH* information exchange within countries or within regions, whether from the perspective of a provider or a recipient of technical and/or financial support.

*any aspect of HRH, including: data or information systems, supply (e.g. headcount, density, domestic production, migration, attrition, retention), demand (consumer and economic), need, accessibility, quality, working conditions (e.g. motivation, working environment, remuneration), management/regulation (e.g. supervision, productivity, performance, efficiency, scope of practice).

**Fig. 1:**
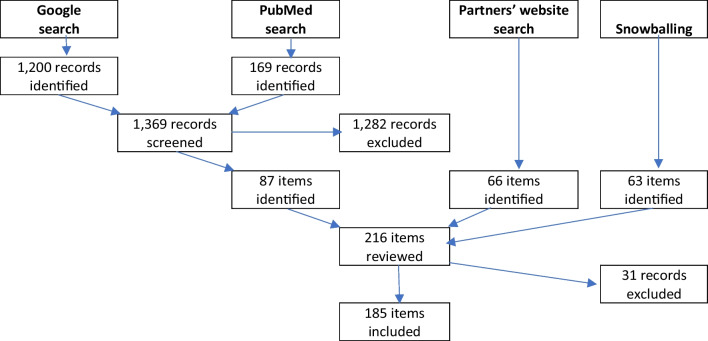
Search results

Of the 1,369 screened items, 87 went forward for full text review. The search of partner websites yielded a further 66 items, and snowballing a further 63. In total, therefore, 216 items were fully reviewed, as summarized in Fig. 1. Of these, 31 were rejected after full review due to not meeting the inclusion criteria. This left 185 that were included in this review.

The included items were reviewed by one researcher, who recorded key pieces of information about each item in an extraction grid, including: document type, partner organization(s) involved, whether the item included information about the organization’s participation in health workforce assessments and/or information exchange (and if so what information was included and whether the document was based on a specific conceptual framework such as a labour market framework). Items were categorized according to the document types listed in Tables 2, 3, 4, 5, which were developed a priori by the authors.

## Findings

### Policies and strategies to guide HRH investments

Effective governance and accountability are more easily achieved with the availability of published policy and strategy documents to guide activities and decisions, which make clear the organization’s strategic priorities. It would be reasonable to expect organizations which are active in the sphere of HRH to provide clear strategic direction on HRH investments in their policies and strategies. This makes it easier to implement policy at a country level, and fosters accountability for the impact of the organization’s activities. Further, the application of a health labour market logic to the development of relevant policy/strategy documents can help organizations to better understand the factors affecting workforce supply and demand at national, regional and global levels, and thus ensure that these documents are comprehensive and relevant [12].

For most partner organizations, the review found at least one policy or strategy document designed to guide the organization’s decisions, including about global health activities. These tend to set out the organization’s overarching vision, values and guiding principles in broad terms. In some cases they specify priority areas for investment, e.g. specific countries, diseases, communities. For about half of the organizations, the review located documents designed to guide decisions about health system strengthening (including HRH). Some of these (e.g. Gavi, Global Fund, UNICEF, UK) explicitly prioritize certain types of HRH investments, in some cases discouraging—but not necessarily ruling out—the payment of recurrent costs such as health worker salaries.

There is widespread recognition among all types of partners that HRH investments are essential for the achievement of global health goals, e.g. Global Fund [13], WHO [7], Japan [14], UK [15], USA [16]. Despite this recognition, policy or strategy documents specifically relating to HRH investments were found for just five organizations: four multilaterals and one bilateral (Table 2). The Global Fund, UNICEF, WHO and USA policy/strategy documents which guide decisions about investments in HRH (bottom row of Table 2), explicitly or implicitly acknowledge the importance of considering investments within a health labour market framework.

**Table 2:** Existence of published documents guiding global health investment decisions

Type of document	Multilaterals identified		Bilaterals identified
	Funding support	Technical support	
Publicly available policy/ strategy/ position statement which guides decisions about global health activities generally	ADB [17]; AfDB [18]; Gavi [19]; GFF [20]; Global Fund [13]	UNFPA [21]; UNICEF [22]; WHO [23]	BMGF [24]; EC [25, 26]; Canada [27, 28] China [29]; France [30–32]; Japan [33, 34]; Norway [35]; USA [36]
Publicly available policy/ strategy/ position statement which guides decisions about investments in health system strengthening (including HRH)	Gavi [37, 38]; GFF [39], Global Fund [40]	UNICEF [41]; WHO [42]	BMGF [43], France [30]; UK [15]; USA [44]
Publicly available policy/ strategy/ position statement which guides decisions about investments in HRH specifically	Gavi [45]; Global Fund [46]	UNICEF [47]; WHO [7]	USA [16, 48]

### Participation in efforts to strengthen health workforce assessments

A health workforce assessment is a process that prepares evidence for decision-makers on strengths, weaknesses, gaps and stakeholders. A workforce assessment may include an “impact assessment”, i.e. an assessment of the potential outcomes and impacts of initiatives and investments, as they relate to the health workforce. When conducted in advance of implementation, health workforce impact assessments represent an attempt to provide a coherent and transparent analysis of the foreseeable effects of a proposed policy or intervention, and thus provide an opportunity to maximize the potential benefits and minimize or offset the potential risks [49].

Health workforce assessments should be considered as part of an organization’s monitoring and evaluation (M&E) activities. The review located a number of documents which described partner organizations’ approach to the M&E of their global health activities, and these are summarized in Table 3. Most partner organizations have published an evaluation policy or strategy document, in recognition of the importance of taking a structured approach to evaluation, and in some cases acknowledging that this has been overlooked in the past. Features of these policy documents which are common to all of the examples located were: a description of how evaluation supports accountability, learning and improvement, and a description of the guiding principles for that organization’s evaluation efforts, such as: transparency, accountability, methodological rigour, and proportionality. Some also specify the types of evaluation which are appropriate for that organization and/or the types of methods to be used. Several explicitly mention the challenges of attributing observed change to the activities of the organization.

**Table 3:** Existence of published documents relating to monitoring and evaluation of global health activities generally, and health workforce assessments specifically

Type of document	Multilaterals identified		Bilaterals identified
	Funding support	Technical support	
Publicly available policy/ strategy/ position statement which guides evaluation of global health investments	EIB [50–52]; Gavi [53]; Global Fund [54, 55]; World Bank [56]	UNFPA [57, 58]; UNICEF [59]; WHO [60]	Australia [61]; BMGF [62]; Germany [63, 64]; Japan [65], Norway [66]; UK [67], USA [68, 69]
Publicly available monitoring and evaluation framework which includes compulsory HRH indicators	–	UNFPA [70]	–
Publicly available monitoring and evaluation framework which includes optional HRH indicators	EIB [52]; Gavi [71, 72]; GFF [73]; Global Fund [55]	UNICEF [41]	–
Public recognition that health workforce impact assessments could be appropriate/helpful	Gavi [74]; Global Fund [46]; World Bank [75]	WHO [76]	–
Publicly available policy to conduct health workforce impact assessments for certain types of activity	–	WHO [7, 10]	–
Publicly available guidance and/or tools about how to conduct health workforce impact assessments	Global Fund [46]	WHO [49, 77, 78]	USA [79, 80]

Table 3 also shows that several multilaterals (but no bilaterals) have published guidance for monitoring HRH indicators as part of M&E efforts. Most of these take a broad health and/or health systems approach, with an HRH component. WHO is the only partner organization to state that it has a policy to conduct health workforce impact assessments as a matter of routine. WHO, Global Fund, and USA have published guidance and/or tools on conducting health workforce impact assessments, all of which are designed around a health labour market framework.

Generally, if one of the stated objectives of an investment is to affect HRH, there is a requirement to monitor and evaluate progress against that objective. Several evaluations of HRH investments were found in this review, e.g. by EIB [81], Global Fund [82], UNFPA [83], UNICEF [84, 85], and Australia [86].^1^ However, if the investment in HRH is part of a broader effort to strengthen health systems or improve health outcomes, M&E efforts tend not to have an explicit focus on HRH.

Where guidance is available on HRH monitoring indicators, it tends to consider outcome and coverage indicators, rather than impacts or implications. Coverage indicators often focus on health worker education and training (e.g. numbers trained, alignment of education curricula with global standards, education programme completion rates), management and supervision (e.g. numbers receiving supportive supervision visits). Outcome indicators often focus on health worker availability (graduate absorption rates, health facility staffing levels and vacancy rates, health worker density and distribution). In addition, the Global Fund has some indicators relating to HRH policy development, the existence of HRH information systems and the conducting of health labour market assessments. Gavi has an indicator to measure the rate at which Gavi-funded positions are transferred into the national health system.

The review found very little acknowledgement among partners of the importance of assessing the indirect or unintended impacts of their activities on the health workforce, and even fewer examples of participation in efforts to strengthen health workforce impact assessments. The few examples were:

- WHO’s development of an ex ante HRH impact assessment tool, which uses a health labour market framework as its basis [49];
- Global Fund’s guidance on which types of HRH investment will be funded, which includes a recommendation (but not a requirement) for a ‘light-touch’ HRH impact assessment at the application stage [46];
- OECD’s work on skills assessment as a method of evaluating the effectiveness of health workforce policies [87, 88].

Although not an impact assessment tool per se, the EIB’s due diligence and monitoring cycle includes a requirement to adhere to its social standards including labour standards and public health [89].

Several partners have embraced the concept of assessing the impact of their activities, and a few could be said to have mainstreamed it as a principle across some or all of their activities, e.g. EIB [90], OECD [91], Germany [92], and USA [93]. Gavi notes that impact assessment can be useful when considering the extent to which observed outcomes and impact can be attributed to the activities of an organization [53]. Table 4 summarizes the types of impact assessment required or recommended by different partners. These results indicate that it is rare for health workforce impact assessments to be required or recommended. Only WHO requires this for technical resolutions and strategies brought before the WHA and WHO Regional Committees [94], and the Global Fund suggests that it might sometimes be helpful when preparing a funding application [46].

**Table 4:** Types of impact assessment required or recommended by partner organizations

Focus topic for impact assessment	Multilaterals identified		Bilaterals identified
	Funding support	Technical support	
Environment and social	EIB [95]; World Bank [96]	OECD [91]; WHO [97]^a^	France [98]; Germany [99]; Japan [100]; Saudi Arabia [101]
Sustainability	–	OECD [91]; WHO [97]^a^	France [102, 103]; Germany [99]; Norway [104]; Saudi Arabia [101]
Development / aid quality or effectiveness	AfDB [105–107]; EIB [108]; Gavi [53]	–	Australia [109]; EC [110]
Gender equality	EIB [111]	OECD [112]	Canada [113]
Health	ADB [114]	WHO [115, 116]	EC [110]
Economy	EIB [117, 118]		Germany [99]
Employment	EIB [119]		EC [110]
Health workforce	Global Fund [46]	WHO [94, 120]	–
Risk mitigation	–	–	Canada [121]; UK [122]^b^
Social	EIB [95]	–	France [98]
Epidemiology	Global Fund [123]	–	–
Infrastructure	World Bank [124]	–	–
Poor and marginalized groups	–	–	Canada [125]
Regulation	–	OECD [126]	–

^a^The original search found an earlier version of this page, which is no longer available, so it was replaced with an updated link

^b^Including risks of unintended consequences

### Participation in efforts to strengthen information exchange

Typically, HRH data are held by numerous organizations using a variety of systems and processes, which can make it difficult to take a broad, labour market approach to policy- and decision-making. The *Global Strategy on HRH* notes that improvements in HRH information architecture and interoperability can address this challenge and thus enable better advocacy, planning, policy-making, governance and accountability [7].

This review found evidence that most partner organizations had participated in efforts to strengthen HRH information exchange, as part of their own activities and/or by providing financial or technical support to other organizations’ activities. The most common “own activity” was being a member of a network whose work included HRH information exchange—nearly all partner organizations did this, regardless of whether the support they provide for the health workforce role is primarily financial or primarily technical. Other “own activities” mostly involved partners whose main role was technical support. These included the publication of analysis of HRH data at a global or regional level, the development or updating of technical guidance or tools, and the development or updating of systems to facilitate data exchange.

The review also located examples of partners providing support to other actors for information exchange activities including: health workforce observatories/HRH planning units, HRH information systems, and supporting the analysis of HRH data for planning purposes at country level (Table 5). The first two types of activity mainly involved financial support, and the third type mainly involved technical support.

**Table 5:** Participation in efforts to strengthen information exchange

Activity type	Activity	Multilaterals identified		Bilaterals identified
		Funding support	Technical support	
Own activities	Membership of a network whose work includes HRH information exchange	Gavi [127]; Global Fund [128]; World Bank [128]	OECD [129–131]; UNFPA [127] WHO [128–132]	BMGF [127]; Canada [127]; EC [133]; France [127] Germany [127]; Japan [127]; Norway [127]; UK [127]; USA [127, 128]
	Contribution to the publication of analysis of HRH data at global or regional level	AfDB [134]; World Bank [135]	OECD [131, 136]; UNFPA [137–140]; WHO [135, 137, 141–147]	BMGF [148]; EC [133, 149]
	Development or updating of technical guidance or tools for HRH information exchange	Global Fund; World Bank [150]	OECD [130]; WHO [120, 150–153]	BMGF [148]; EC [154], Germany [155]; USA [156, 157]
	Development or updating of a system to facilitate HRH information exchange	World Bank [158]	OECD [136, 159–161];WHO [151, 162]	–
Financial or technical support for others’ activities	Establishment or strengthening of an organization or department with a focus on HRH, e.g. a health workforce observatory or HRH planning unit	EIB [163]; Global Fund [46]; World Bank [163]	WHO [164–168]	EC [163, 168]; Norway [163]; UK [163]; USA [164, 169, 170]
	Establishment or strengthening of HRH information systems or data platforms	EIB; Global Fund [171]	WHO [172–175]	BMGF [176]; Canada [177]; UK [178, 179]; USA [169, 180–184]
	Supporting the analysis of HRH data for planning purposes at country level, e.g. health labour market analysis or HRH needs assessments	Global Fund [46]	OECD [136, 159, 160]; WHO [185–187]	BMGF; EC [154]; USA [188]

Descriptions and examples of each type of information exchange activity are shown below.

#### Membership of a network whose work includes HRH information exchange

Networks such as the Global Health Workforce Network and the OECD/WHO/International Labour Organization ‘Working 4 Health’ programme have brought partner organizations together with a view to improving harmonization and efficiency. The Global Health Workforce Network’s activities included the development and promotion of systems and tools for harmonization of indicators, data collation and reporting systems and tools [132]. The ‘Working 4 Health’ programme had a specific action to institute an interagency data exchange mechanism [129].

#### Contribution to the publication of analysis of HRH data at a global or regional level

Partner organizations have identified the need for publications to highlight global or regional patterns and gaps in the available data and evidence which can support policy dialogue. For example, WHO has published analysis including: the *State of the World’s Nursing 2020* report [142], gender issues affecting health workers [143], and the impact of COVID-19 on health workers [144]. Similarly, UNFPA led the third *State of the World’s Midwifery 2021* report, including data on midwives and other health workers [137]. UNFPA has also published regional midwifery workforce reports [138–140]. Other publications have emerged as a result of governing bodies’ resolutions, e.g. WHO’s monitoring of the effectiveness of the *Global Code of Practice on the International Recruitment of Health Personnel* [141].

#### Development or updating of technical guidance or tools for HRH information exchange

Partners have developed and shared good practice guidance to improve the quality of HRH information systems, and tools to support the use of HRH data for planning and forecasting. For example, the USA funded the production of standards and best practices for health information systems, including a specific HRH module [156], and the development of an HRH Action Framework with a section on information systems [157].

Other partner activities included tools to reduce the reporting burden on countries, e.g. the OECD/Eurostat/WHO European Region Joint Questionnaire collects data from multiple countries on HRH indicators, including international mobility of health workers [130, 131].

#### Development or updating of a system or platform to facilitate HRH information exchange

Several partners have contributed to efforts to make global and regional reporting on HRH data easier, and to base reporting on international standards. For example, WHO led the development and progressive implementation of National Health Workforce Accounts (NHWA). NHWA is designed to: support health workforce monitoring through a system strengthening approach, avoid duplication of effort, and support the development of global and regional health workforce reports. NHWA data are publicly accessible via its data portal [151].

OECD advises member countries on HRH planning and management, including the collation and publication of key HRH statistics to enable cross-country comparison [136, 159, 160], and EC publishes education statistics, including data on education of health workers [189].

#### Establishment or strengthening of an organization or department with a focus on HRH, e.g. a health workforce observatory or HRH planning unit

In recognition of the need in some countries to build capacity for using HRH data effectively for planning and forecasting, partners have supported the establishment or strengthening of HRH observatories and planning units within ministries of health. For example, the Global Fund funded positions in an HRH planning unit in Liberia [46], and the USA has supported the strengthening of HRH observatories in countries including Ghana and Jordan [169, 170].

#### Establishment or strengthening of HRH information systems or data platforms

Activities in this category include attempts to share good practice and ensure a multi-sectoral approach to health and health systems. For example, BMGF provided financial support to the WHO UHC compendium—a global repository of interventions for UHC, including defining workforce requirements for health interventions [190]. They also include efforts to improve the coverage and interoperability of HRH information systems (HRHIS). For example, BMGF supported a multi-country assessment of HRHIS to identify common challenges and opportunities for improvement [176]. Canada has funded HRHIS strengthening in Bangladesh [177]; the UK in countries including the Democratic Republic of Congo [178] and Nepal [179], and the USA in countries including Kenya, Uganda, and Zambia [182, 183]. The USA funds the HRH2030 programme, the activities of which include improving HRHIS [180]. HRH2030 has supported technical assistance for NHWA in Ethiopia, Indonesia and the Philippines [181].

#### Supporting the analysis of HRH data for planning purposes at country level, e.g. health labour market analysis or HRH needs assessments

In countries with gaps in national capacity for HRH data collection, collation and analysis, partners have provided technical support. For example, The Global Fund has supported HRH inventories in Eswatini and Lesotho and a health workforce gap analysis in Sierra Leone [46]. It also supported technical assistance to Chad, Democratic Republic of Congo, Mali, Niger, and Nigeria to improve the use of HRH data for policy and decision-making [171] The World Bank supports countries to conduct data collection and analysis to inform HRH policy and decision-making [158]. WHO has provided technical assistance for health labour market analyses for individual countries including: Bangladesh [185], India [186], and Sri Lanka [187], the findings of which have informed policy dialogue and investment decisions by national governments other development partners.

## Discussion

This study found that it is not standard practice for partner organizations to include a requirement or recommendation to conduct health workforce assessments as a matter of routine, nor to recommend assessment of the impact of their activities. However, this study did locate evidence that a considerable number of partner organizations have embraced the concept of assessing the impact of their activities on other areas. Where partners require or recommend a specific type of impact assessment, it is usually in an area relating to one of their areas of strategic focus, e.g. Canada has a strategic focus on gender equality and therefore requires a gender equality assessment. This suggests that efforts to mainstream health workforce impact assessments will be more successful within partner organizations which have (and publicly acknowledge) a specific strategic focus on HRH. This underlines the importance of having policy or strategy documents which acknowledge that HRH is a strategic focus and which guide decisions about HRH activities and investments.

Despite the widespread acknowledgement of the importance of HRH for global health initiatives and the recent increase in development assistance for the health workforce [191], and despite some external commentators calling for global health partners to focus more on HRH issues [192–194], we found few examples of specific guidance on HRH investments. Even among partners who acknowledge that HRH are a vital investment, there can be a lack of specificity about their own HRH roles and responsibilities, which can lead to confusion when organizing technical cooperation at country level [195]. Some partners have taken action in response to this issue, e.g. GFF and UNICEF have recently included specific reference to their approach to HRH investments for the first time in their policy documents [38, 195, 196]. All organizations involved in global health initiatives should consider whether their current policy and strategy documents contain sufficient detail to effectively guide their activities as they relate to the health workforce.

The openness of many partner organizations to the principle of impact assessment may represent an opportunity to expand this into HRH, e.g. by adding an HRH module to an existing M&E framework and/or impact assessment tool. Calls for the mainstreaming of impact assessments have been heard since 2002 [197]. In some cases, lack of action may be at least partly due to gaps in M&E capacity [198]. In others, it may stem from a lack of recognition that effective HRH leadership requires tools that support understanding of complex situations, especially in challenging times such as global pandemics [5]. We found only a few examples of efforts to strengthen health workforce impact assessments, of which only the Global Fund and WHO tools are based on an established labour market framework.

This study found that most of the focus organizations have participated actively in HRH data exchange activities. This indicates a high level of recognition that harmonization of the collection, collation and use of HRH data is vital for better advocacy, planning, policy-making, governance, accountability, and has the potential to reduce the reporting burden on countries. The results of these efforts can be seen in many of the publications referenced in this paper, and some more recent publications [199], but even the more recent global reports on the health workforce include caveats about the availability and quality of data from many countries [137, 142]. This indicates the need for additional efforts on HRH information exchange as well as on health workforce assessment.

This study was subject to a number of limitations. First, the searches were conducted in English, so will have missed relevant documents published in other languages. Second, although structured, the search was not fully systematic, so could have overlooked relevant documents. Third, it is possible that relevant documents exist internally within organizations but are not publicly available, and therefore will not have been included in this review.

The shortcomings identified in this study indicate the need for additional action from partner organizations. Partners whose main role is the provision of funding support should adapt their policies to allow greater, more predictable, sustainable investment in the health workforce. In countries where the macroeconomic situation and health labour market conditions warrant it, this means shifting the focus away from short-term in-service training and disease-specific incentives to allow investments in pre-service education and jobs for general service staff [7]. Financing the creation of new jobs in the health sector, with decent working conditions, should be recognized as a productive investment, with potential returns for health, employment, economic growth and the economic empowerment of women and youth [9].

Consideration should also be given to reorienting existing multilateral and bilateral funding facilities to support international investment in health systems (including HRH) which adhere to the principles of aid effectiveness and align with other relevant architecture at global and national levels. The response to the COVID-19 pandemic demonstrated that the capacity exists to increase resource mobilization for HRH, but that opportunities remain for greater alignment of this support to national HRH priorities and mechanisms [191]. Regional partners can play an important advocacy role to help generate the political will to support HRH investments. Mechanisms should be established to allow reliable and consistent monitoring of HRH investment.

Partners whose main role is to provide technical support should establish governance mechanisms to ensure that their planned interventions include an assessment of health workforce implications and consider sustainability by addressing the underlying causes of HRH shortages [49].

## Conclusions

Although there is evidence of participation in efforts to strengthen health workforce assessments and (especially) information exchange, the achievement of the *Global Strategy’s* fourth strategic objective requires more structured policies for the monitoring and evaluation of health workforce investments to optimize the value of these investments and contribute towards achieving global and national health goals.

## Supplementary Information

**Additional file 1.** Search terms.

## Data Availability

Not applicable.

## Abbreviations

ADB: Asian Development Bank
AfDB: African Development Bank
BMGF: Bill and Melinda Gates Foundation
ComHEEG: United Nations Secretary-General’s High-Level Commission on Health Employment and Economic Growth
EC: European Commission
EIB: European Investment Bank
GFF: Global Financing Facility
HRH: Human resources for health
HRHIS: Human resources for health information system
M&E: Monitoring and evaluation
NHWA: National Health Workforce Accounts
OECD: Organization for Economic Cooperation and Development
PAHO: Pan-American Health Organization
UHC: Universal health coverage
UK: United Kingdom
UNFPA: United Nations Population Fund
UNICEF: United Nations Children’s Fund
USA: United States of America
WHA: World Health Assembly
WHO: World Health Organization

## Footnotes

1 This is almost certainly not an exhaustive list: some partner organizations’ websites included repositories of thousands of evaluation reports and in the time available it was not possible to look at every one of them. It was sometimes possible to search for specific types of report using keywords, but not always.
