# Supplementary material for: The roles and involvement of global health partners in the health workforce: an exploratory analysis
Source: Hum Resour Health. 2023 May 24;21:41. doi: 10.1186/s12960-023-00825-5 (PMC10208191; doi:10.1186/s12960-023-00825-5)
Supplement: Supplementary file 1 — Additional file 1. Search terms. [file 12960_2023_825_MOESM1_ESM.docx]

**Annex: Search terms**

*Grey literature*

In total, 24 Google searches were performed, 23 of which consisted of the names of the 23 partner organizations plus the following search terms:

(“human resources for health” OR “health workforce” OR “health workers” OR “health occupations” OR “health labour market” OR “HRH” OR “health system”) AND (policy OR strategy OR “technical guidance” OR “technical note” OR assessment OR measurement OR impact OR data OR aid OR development OR assistance OR harmonization).

The above searches found relatively little information about data exchange, so a 24^th^ Google search was performed using the following search terms: “human resources for health observatory” OR “health workforce observatory” OR human resources for health information system” OR “health labour market analysis”.

In addition, the search function of the 23 partner organizations’ websites was used to find any additional references to “impact assessment”, “human resources for health”, “health workforce”, “data exchange”, “health workforce observatory”, “human resources for health observatory”, “human resources information system” or “information network”.

*Peer reviewed literature:*

The peer-reviewed literature search was conducted on 7 June 2021 using the PubMed search engine, with the following filters and search terms:

*Publication date*: 1/11/16 to 7 June 2021
*Type*: comment, editorial, evaluation study, government publication, guideline, historical article, journal article, letter, news, newspaper article, technical report
*Species*: Humans
*Language*: English, French, Spanish
*Search terms*: ("GAVI"[Title/Abstract] OR "Global Fund"[Title/Abstract] OR "United Nations"[Title/Abstract] OR "UNFPA"[Title/Abstract] OR "UNICEF"[Title/Abstract] OR "World Health Organization"[Title/Abstract] OR "OECD"[Title/Abstract] OR "World Bank"[Title/Abstract] OR "development bank"[Title/Abstract] OR “European Investment Bank”[Title/Abstract] OR “Global Financing Facility” OR "Gates Foundation"[Title/Abstract] OR "BMGF"[Title/Abstract] OR "European Commission"[Title/Abstract] OR "Government"[Title/Abstract] OR "observatory"[Title/Abstract]) AND ("Health Workforce"[MeSH Major Topic] OR "Staff Development"[MeSH Major Topic] OR "Workload"[MeSH Major Topic] OR "Personnel Staffing and Scheduling"[MeSH Major Topic]) AND (policy OR strategy OR "impact assessment" OR "measurement" OR "data" OR "aid" OR "development" OR "assistance" OR “harmonization”)

A second search was run on 22 June 2021 with the following filters and search terms:

*Publication date*: 1/11/16 to 22 June 2021
*Type*: comment, editorial, evaluation study, government publication, guideline, historical article, journal article, letter, news, newspaper article, technical report
*Species*: Humans
*Language*: English, French, Spanish
*Search terms*: ("GAVI"[Title/Abstract] OR "Global Fund"[Title/Abstract] OR "United Nations"[Title/Abstract] OR "UNFPA"[Title/Abstract] OR "UNICEF"[Title/Abstract] OR "World Health Organization"[Title/Abstract] OR "OECD"[Title/Abstract] OR "World Bank"[Title/Abstract] OR "development bank"[Title/Abstract] OR "European Investment Bank"[Title/Abstract] OR “Global Financing Facility[Title/Abstract]” OR "Gates Foundation"[Title/Abstract] OR "BMGF"[Title/Abstract] OR "European Commission"[Title/Abstract] OR "Government"[Title/Abstract] OR "observatory"[Title/Abstract]) AND ("Health Workforce"[MeSH Major Topic] OR "Staff Development"[MeSH Major Topic] OR "Workload"[MeSH Major Topic] OR "Personnel Staffing and Scheduling"[MeSH Major Topic]) AND ("information system" OR "observatory" OR "information network")
